# B.1.526 SARS-CoV-2 Variants Identified in New York City are Neutralized by Vaccine-Elicited and Therapeutic Monoclonal Antibodies

**DOI:** 10.1128/mBio.01386-21

**Published:** 2021-07-27

**Authors:** Hao Zhou, Belinda M. Dcosta, Marie I. Samanovic, Mark J. Mulligan, Nathaniel R. Landau, Takuya Tada

**Affiliations:** a Department of Microbiology, NYU Grossman School of Medicine, New York, New York, USA; b NYU Langone Vaccine Center and Department of Medicine, NYU Grossman School of Medicine, New York, New York, USA; Columbia University College of Physicians & Surgeons

**Keywords:** SARS-CoV-2, neutralization, B.1.526, spike protein, Pfizer BNT162b2, Moderna mRNA-1273, REGN10933, REGN10987

## Abstract

DNA sequence analysis recently identified the novel SARS-CoV-2 variant B.1.526 that is spreading at an alarming rate in the New York City area. Two versions of the variant were identified, both with the prevalent D614G mutation in the spike protein, together with four novel point mutations and with an E484K or S477N mutation in the receptor-binding domain, raising concerns of possible resistance to vaccine-elicited and therapeutic antibodies. We report that convalescent-phase sera and vaccine-elicited antibodies retain full neutralizing titer against the S477N B.1.526 variant and neutralize the E484K version with a modest 3.5-fold decrease in titer compared to D614G. The E484K version was neutralized with a 12-fold decrease in titer by the REGN10933 monoclonal antibody, but the combination cocktail with REGN10987 was fully active. The findings suggest that current vaccines and Regeneron therapeutic monoclonal antibodies will remain protective against the B.1.526 variants. The findings further support the value of widespread vaccination.

## OBSERVATION

Severe acute respiratory syndrome coronavirus 2 (SARS-CoV-2) is a highly transmissible and pathogenic coronavirus that became an ongoing pandemic late in 2019. Infection rates have begun to fall, at least in part, due to large-scale vaccination efforts. In addition, treatment of infected patients with monoclonal antibodies against the spike protein have been found to reduce hospitalization and mortality. The recent emergence of SARS-CoV-2 variants raises concerns with regard to vaccine efficacy and the effectiveness of monoclonal antibody therapy. The vast majority of sequenced SARS-CoV-2 isolates contain a D614G mutation in the spike protein (1) that increases viral infectivity and transmissibility (2–4) and, subsequently, variants with multiple mutations in the spike protein and enhanced transmissibility have emerged in the United Kingdom (5, 6), South Africa (7), Brazil (8), and the United States (9), raising concerns of diminished neutralization by immune sera-elicited antibodies and escape from therapeutic monoclonal antibodies.

Recent reports have identified a novel variant in New York City termed B.1.526 that was rapidly spreading (10–12). The variant was identified in November 2020; by January 2021, the variant accounted for 5% of genomes sequenced from individuals in New York and by mid-February was detected with a frequency of 12.3% (10–12). The variant contains several mutations in the spike protein, some of which have not been found in previous variants. Two versions of B.1.526 were identified, both having the D614G and A701V mutations and, in addition, the mutations L5F, T95I, and D253G, which are not present in previously reported variants. One version of B.1.526 also contains the E484K mutation, which is present in the B.1.351 and B.1.1.248 variant spike proteins and allows for partial escape from immune serum neutralization (13–16); the other lacks the E484K mutation but has a nearby S477N mutation, which lies within the receptor binding domain (RBD) and thus may influence affinity for the entry receptor ACE2. The D253G mutation is located in the amino-terminal supersite that serves as a binding site for neutralizing antibodies, while A701V is located adjacent to the furin processing site. The combination of mutations raises concerns that the B.1.526 variant might evade vaccine-elicited and therapeutic antibodies.

Previous studies have shown that the E484K mutation in the B.1.351 spike protein leads to a degree of resistance to neutralization by both infection- and vaccine-elicited antibodies, as well as to the REGN10933 therapeutic monoclonal antibody (17–19). Moreover, the B.1.351 variant spike protein has been found to reduce the level of protection provided by vaccination in populations in which the variant has become prevalent (17, 20).

In this study, we determined the susceptibility of the B.1.526 variants to neutralization by convalescent-phase sera and sera from individuals vaccinated with the Pfizer BNT162b2 and Moderna mRNA-1273 vaccines, and by the Regeneron therapeutic monoclonal antibodies (18, 21). We found that the B.1.526 variant (S477N) was fully susceptible to neutralization, while the B.1.526 variant with the E484K mutation neutralized with a modest (3.5-fold) reduction in titer by convalescent and vaccine-elicited antibodies. The B.1.526 spike proteins were readily neutralized by the Regeneron antibody cocktail.

**FIG 1:**
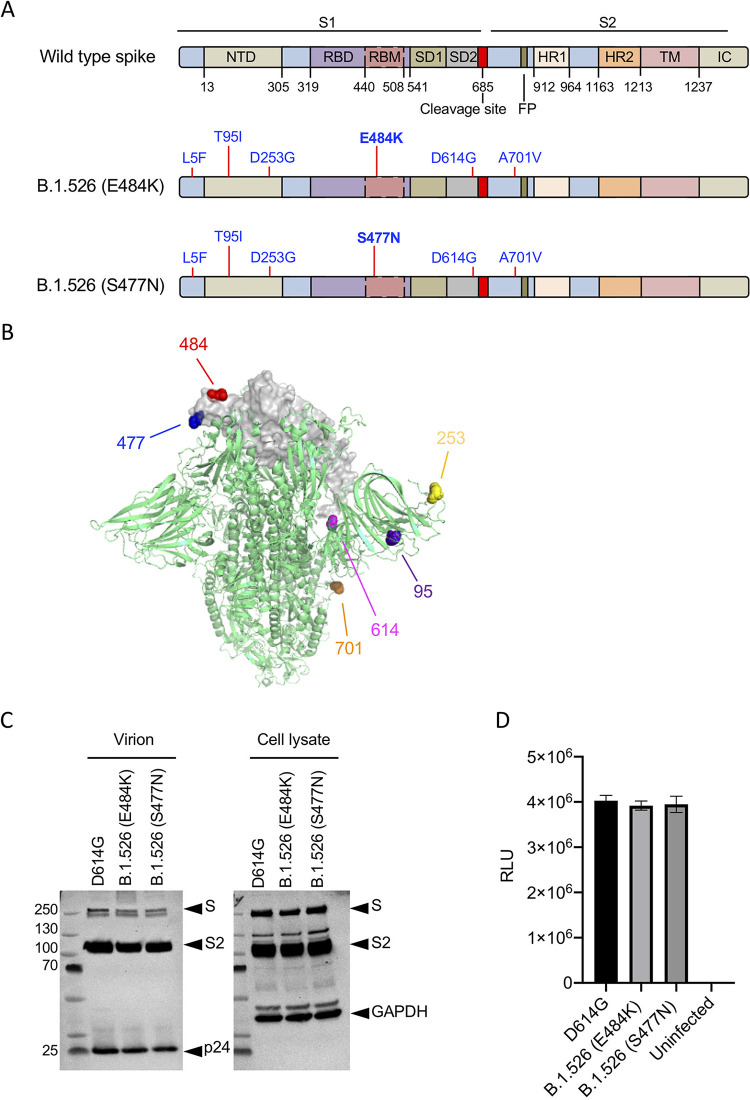
Analysis of B.1.526 pseudotyped lentiviral virions. (A) The domain structure of the wild-type SARS-CoV-2 spike protein is shown above. NTD, N-terminal domain; RBD, receptor-binding domain; RBM, receptor-binding motif; SD1 subdomain 1; SD2, subdomain 2; FP, fusion peptide; HR1, heptad repeat 1; HR2, heptad repeat 2; TM, transmembrane region; IC, intracellular domain. The locations of the mutations in the two B.1.526 spike proteins are diagrammed below with the distinguishing E484K and S477N mutations in bold. (B) The locations of the B.1.526 variant spike protein mutations are shown on the 3D structure of the trimeric spike protein. One RBD region is shown for simplicity. The 484 (red) and 477 (blue) amino acid residues are indicated. (C) Immunoblot analysis of B.1.526 spike protein pseudotyped lentiviral virions and cell lysates of transfected producer cells. The blots were probed for the full-length S and processed S2 proteins and with anti-P24 antibody to detect the virions. GAPDH served as a loading control for the cell lysates. Arrows indicate the full-length spike (S), S2 subunit (S2). (D) Infectivity of B.1.526 pseudotyped virus in ACE2.293T cells. ACE2.293T cells were infected with pseudotyped viruses normalized for RT activity. Luciferase activity was measured 2 days postinfection as relative light units (RLU).

The B.1.526 variant spike proteins contain the D614G mutation, a shared set of novel mutations (L5F, T95I, D253G, and A701V), and either E484K or S477N, both of which lie within the RBD (Fig. 1A and B). To study the B.1.526 spike proteins, we constructed spike protein expression vectors for both B.1.526 versions and used these to produce lentiviral pseudotype reporter viruses as previously described. Immunoblot analysis showed that both B.1.526 spike proteins were expressed and processed in transfected human 293T cells and that both were incorporated into virions at a level comparable to that of the wild-type (D614G) spike protein (Fig. 1C). The infectivity of B.1.526 variant pseudotypes on ACE2.293T was similar to that of wild type (Fig. 1D).

**FIG 2:**
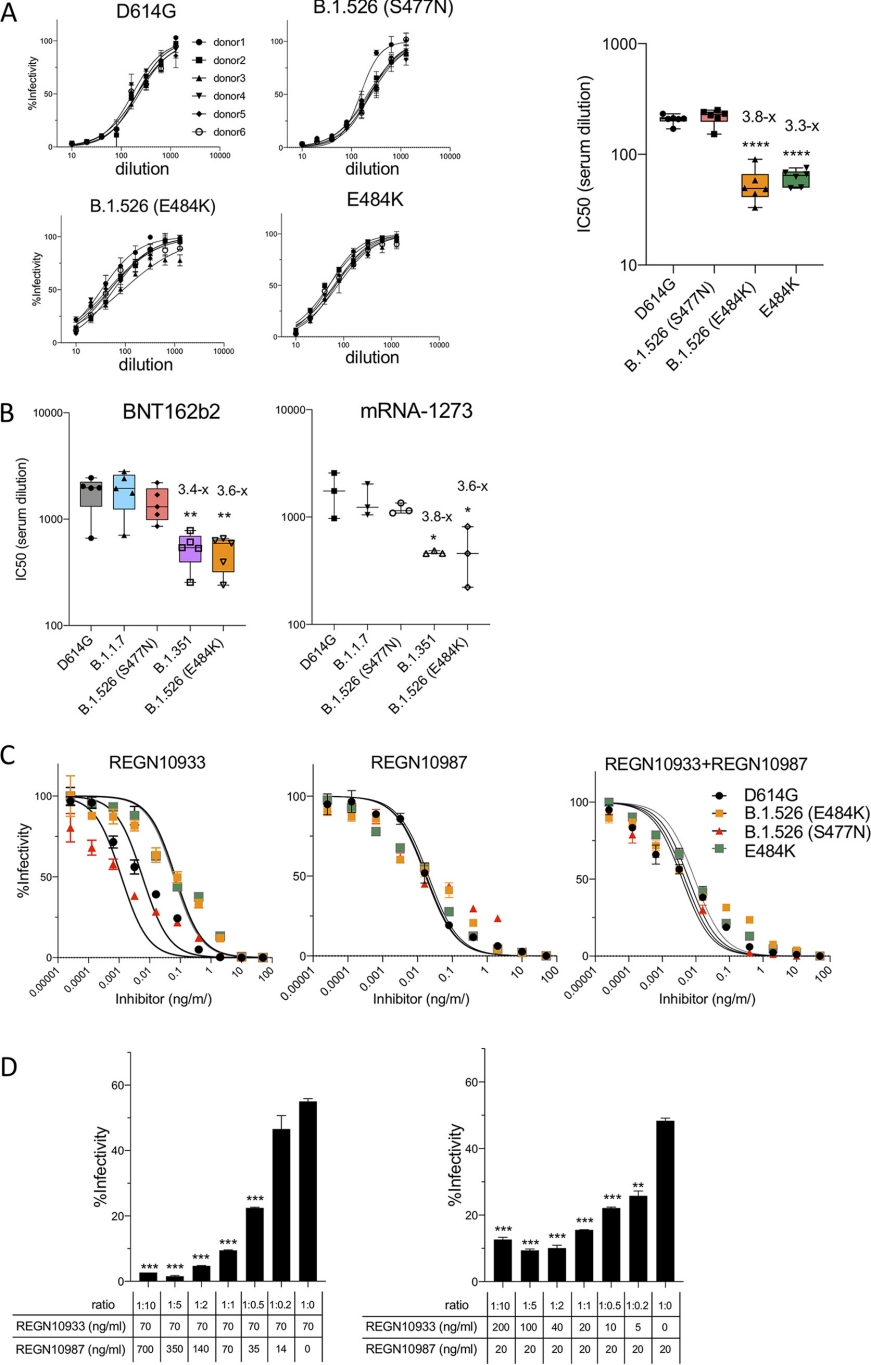
Convalescent serum, antibodies elicited by BNT162b2 vaccine, antibodies elicited by mRNA-1273 vaccine, antibody REGN10933, and antibody REGN10987 all neutralize B.1.526 variant spikes. (A) Neutralization of viruses with D614G, B.1.526 variant spikes by convalescent-phase sera (*n* = 6). The data are shown as the percentage infectivity in the absence of serum (left). IC_50_ (serum dilution) of sera from convalescent individuals (*n* = 6) against virus with B.1.526 and E484K variant spikes (right). (B) Neutralizing titers of serum samples from BNT162b2-vaccinated individuals (*n* = 5) (left) and mRNA-1273-vaccinated donors (*n* = 3) (right) were measured. IC_50_ values of neutralization of virus with D614G, B.1.1.7, B.1.351, and B.1.526 are shown. (C) A 1:1 mixture of the two antibodies was measured on viruses pseudotyped with B.1.526, D614G and E484K variant spike proteins. Neutralization curves of REGN10933, REGN10987, and a 1:1 mixture of REGN10933 and REGN10987 on viruses with the B.1.526, D614G and E484K variant spike proteins. (D). The effect of the MAb ratio on neutralization of variant B.1.526 (E484K) was tested by holding REGN10933 constant at its IC_50_ and titrating in REGN10987 (left) or holding REGN10987 constant at its IC_50_ and titrating in REGN10933.

To test the ability of convalescent-phase sera to neutralize the B.1.526 viruses, we determined the neutralizing antibody titers of sera from individuals who had been infected prior to April 2020 on viruses with D614G, B.1.526 and E484K spike proteins (Fig. 2A). The results showed that neutralizing titers against the S477N B.1.526 variant were similar to that of D614G, while the neutralizing titers against the E484K B.1.526 variant decreased by 3.8-fold, a modest decrease that was attributed to the E484K mutation.

To determine the ability of vaccine-elicited antibodies to neutralize the B.1.526 viruses, we determined neutralizing titers of serum specimens from individuals vaccinated with Pfizer BNT162b2 or Moderna mRNA-1273 vaccines. The results showed that BNT162b2 vaccine serum-elicited antibodies neutralized the D614G and B.1.1.7 viruses with similarly high titers, while titer for neutralization of B.1.351 was decreased by 3.4-fold (Fig. 2B, left). Analysis of the B.1.526 titers showed the S477N version was neutralized with a titer similar to D614G; neutralization titers of the E484K version were decreased by 3.6-fold, a titer similar to that of B.1.351. Neutralization by sera elicited by the Moderna vaccine showed a very similar pattern (Fig. 2B, right).

Analysis of the Regeneron monoclonal antibodies showed that REGN10987 neutralized both B.1.526 variants with no loss of titer (Fig. 2C, Table 1). REGN10933 neutralized virus with the S477N B.1.526 spike protein with a high titer but was 12-fold less active against the E484K B.1.526 version (Fig. 2C, Table 1). The REGN-COV2 cocktail potently neutralized the B.1.526 spike variants despite the partial loss of neutralizing activity against the E484K version of B.1.526. While the two monoclonal antibodies do not have overlapping binding sites, it appeared they may have some synergistic effect when combined. To test this possibility, we held one antibody constant at its 50% inhibitory concentration (IC_50_) and then titrated in the other antibody. The results showed that when REGN10987 was titrated in, it neutralized the virus efficiently but that, conversely, when REGN10933 was titrated in, the virus could not be completely neutralized (Fig. 2D). This result suggests that REGN10933 binding may act to make the REGN10987 epitope more accessible.

**TABLE 1:** The IC_50_ values of the monoclonal antibodies and the combination cocktail on viruses with the variant spike proteins as calculated using the data in Fig. 2C

Variant	IC_50_ (ng/ml)		
	REGN10933	REGN10987	REGN10933+REGN10987
D614G	5.7	18.1	4.7
B.1.526 (E484K)	69.4	20.6	6.2
B.1.526 (S477N)	1.2	17.2	3.9
E484K	62.4	16.7	9.2

The recently identified B.1.526 variant SARS-CoV-2 appears to be increasing in prevalence in New York City, raising concerns about reinfection and immunoevasion (10–12). We report here that both S477N and E484K versions of B.1.526 were neutralized well by convalescent and vaccine-elicited antibodies. The E484K version of B.1.526 did show a significant, nearly 4-fold, decrease in neutralization by vaccine-elicited antibodies, but this represents a modest decrease in titer that is not expected to result in a significant decrease in the protection provided by vaccination and is not expected to result in an increased susceptibility to reinfection. The S477N version was neutralized with no decrease in titer. As we reported, neutralizing antibody titers determined by pseudotyped virus assay closely reflect those measured by live SARS-CoV-2 assay (22).

Our results showed that REGN10987, which binds to the side of the RBD (18, 21), maintains potent neutralizing activity against both versions of B.1.526 but that REGN10933, which binds to the top face of the RBD, that interacts with ACE2, loses 12-fold potency against the E484K version. The decrease in neutralizing titer was caused by the E484K mutation and is similar to the previously reported loss of titer against B.1.351, which also bears the mutation (17–20, 23–25). Despite the partial loss of activity by REGN10933 against B.1.526, the neutralizing activity of the combined antibody cocktail remained high.

Our findings should assuage concerns that the B.1.526 variant will evade protection provided by vaccine-elicited antibodies and suggest that Regeneron therapeutic antibody therapy will retain its effectiveness against the variant. Nevertheless, B.1.526 appears to be spreading at an alarming rate, demonstrating the value of widespread vaccination efforts.

### Plasmids.

pLenti.GFP.NLuc dual green fluorescent protein (GFP)/nanoluciferase lentiviral vector, pcCOV2.Δ19S codon-optimized SARS-CoV-2 spike gene expression vector, HIV-1 Gag/Pol expression vector pMDL, and HIV-1 Rev expression vector pRSV.Rev have been previously described (26). B.1.526 spike mutations were introduced into pcCOV2.Δ19S by overlap extension PCR. All plasmid sequences were confirmed by DNA nucleotide sequencing.

### Cells.

293T cells were cultured in Dulbecco’s modified Eagle medium (DMEM) supplemented with 10% fetal bovine serum (FBS) and 1% penicillin/streptomycin (P/S) at 37°C in 5% CO_2_. ACE2.293T is a clonal cell line that stably expresses a transfected human ACE2. The cells were maintained in DMEM/1 μg/ml puromycin/10% FBS/1% P/S.

### Human sera and monoclonal antibodies.

Convalescent sera and sera from BNT162b2- or Moderna-vaccinated individuals were collected on day 7 following the second immunization at the NYU vaccine center with written consent under IRB approval (IRB 20–00595 and IRB 18–02037). Donor age and gender were not reported. Regeneron monoclonal antibodies (REGN10933 and REGN10987) were prepared as previously described (26).

### SARS-CoV-2 spike lentiviral pseudotypes.

SARS-CoV-2 spike-pseudotyped lentiviruses were produced by cotransfection of 293T cells with pMDL, pLenti.GFP-NLuc, pcCoV2.S-Δ19 (or variant spikes), and pRSV.Rev, as previously described (26). The viruses were concentrated by ultracentrifugation and normalized by reverse transcriptase (RT) activity. To quantify neutralizing antibody, sera were serially diluted 2-fold and then incubated with pseudotyped virus (approximately 2.5 × 10^7^ cps) for 30 min at room temperature. The mixture was then added to 1 × 10^4^ ACE2.293T cells, corresponding to a multiplicity of infection (MOI) of 0.2 in a 96-well cell culture dish. After 2 days, luciferase activity was measured using Nano-Glo luciferase substrate (Nanolight). Luminescence was read in an Envision 2103 microplate luminometer (PerkinElmer).

### Immunoblot analysis.

Cells were lysed in buffer containing 50 mM HEPES, 150 mM KCl, 2 mM EDTA, 0.5% NP-40, and protease inhibitor cocktail. Lysates (40 μg) were separated by SDS-PAGE and transferred to a polyvinylidene difluoride membrane. The membranes were probed with anti-spike monoclonal antibody (MAb) (1A9) (GeneTex), anti-p24 MAb (AG3.0), and anti-GAPDH MAb (Life Technologies) followed by goat anti-mouse horseradish peroxidase (HRP)-conjugated secondary antibody (Sigma). The blots were visualized with luminescent substrate (Millipore) and quantified on an iBright CL1000 imager.

### Quantification and statistical analysis.

All experiments were performed in technical duplicates or triplicates and data were analyzed using GraphPad Prism 8. Statistical significance was determined by the two-tailed unpaired *t* test. Significance was based on two-sided testing and attributed at *P* < 0.05. Confidence intervals are shown as the mean ± standard deviation (SD) or standard error of the mean (SEM). (*, *P* ≤ 0.05; **, *P* ≤ 0.01; ***, *P* ≤ 0.001; ****, *P* ≤ 0.0001). The PDB file of D614G SARS-CoV-2 spike protein (7BNM) was downloaded from the Protein Data Bank. A 3D view of the protein was obtained using PyMOL.
